# Physiological Approach to the Use of the Natural Compound Quinate in the Control of Sensitive and Resistant *Papaver rhoeas*

**DOI:** 10.3390/plants9091215

**Published:** 2020-09-16

**Authors:** Ana Zabalza, Ainhoa Zulet-González, Maria Barco-Antoñanzas, Mikel V. Eceiza, Miriam Gil-Monreal, Mercedes Royuela

**Affiliations:** Institute for Multidisciplinary Research in Applied Biology (IMAB), Universidad Pública de Navarra, Campus Arrosadia s/n, 31006 Pamplona, Spain; ana.zabalza@unavarra.es (A.Z.); ainhoa.zulet@unavarra.es (A.Z.-G.); maria.barco@unavarra.es (M.B.-A.); mikel.vicente@unavarra.es (M.V.E.); mirian.gil@unavarra.es (M.G.-M.)

**Keywords:** quinate, corn poppy, shikimate pathway, physiological effects, sulfonylureas, free amino acids

## Abstract

Quinate (1,3,4,5-tetrahydroxycyclohexanecarboxylate) is a compound synthesized in plants through a side-branch of the shikimate biosynthesis pathway, which is accumulated after glyphosate and acetolactate synthase inhibiting herbicides (ALS-inhibitors) and has phytotoxic potential. The objective of this study was to evaluate the phytotoxicity of quinate on several weed species. Among the species evaluated, *Cynodon dactylon*, *Bromus diandrus*, *Lolium rigidum*, *Sinapis alba*, and *Papaver rhoeas*, *P. rhoeas* was the most sensitive, and its growth was controlled with quinate concentrations above 100 mM at the phenological stage of 6–8 true leaves. A physiological study, including the shikimate pathway and the physiological markers of ALS-inhibitors (carbohydrates and amino acids), was performed in the sensitive and resistant plants treated with sulfonylureas or quinate. The typical physiological effects of ALS-inhibitors were detected in the sensitive population (free amino acid and carbohydrate accumulation) and not detected in the resistant population. The mode of action of quinate appeared to be related to general perturbations in their carbon/nitrogen metabolism rather than to specific changes in the shikimate pathway. These results suggest the possibility of using quinate in the weed control management of *P. rhoeas*.

## 1. Introduction

Two groups of herbicides that inhibit amino acid biosynthesis are among the most important herbicidal modes of action in global market value [1]. One of them is glyphosate, whose target site is the 5-enolpyruvylshikimate 3-phosphate synthase (EPSPS) in the aromatic amino acid biosynthetic pathway [2]. The other group is acetolactate synthase (ALS) inhibitors, where different active ingredients belonging to different chemical classes share ALS as the target site, which is in the biosynthetic pathway of branched-chain amino acids [3]. Although the target site of each herbicide group is different, several physiological effects common to both glyphosate and ALS-inhibitors have been reported, including a general increase in the total free amino acids [4] and carbohydrate content [5,6]. Another common effect is the accumulation of quinate in the leaves of plants treated with both types of herbicides [7]. Quinate (1,3,4,5-tetrahydroxycyclohexanecarboxylic acid) is formed in a branch pathway of the shikimate pathway, and it is considered a reserve compound of the pathway [8].

Overreliance on chemical weed control has led to a rise in herbicide-resistant weed populations [9]. Today, weed resistance is a rapidly growing global problem with 262 different species with at least one population resistant to one group of herbicides with the same site of action; weeds have also evolved resistance to 23 of the 26 known herbicide sites of action [10]. This global resistance problem is increasingly difficult to solve, with few new herbicides with unique modes of action to counter this trend and often no economical alternatives to herbicides in large-acreage crops. In this context, it is very important to discover and develop new effective and economical herbicides with different mechanisms of action [11]. Progress on this issue has experienced a renewed interest in this research field and the development programs observed in the agrichemical industry, academic, and governmental institutions [12]. A specific emphasis on natural products from plants (allelochemicals) and microorganisms that could lead to new chemistries and bioherbicides is being developed [11].

Both ALS and EPSPS inhibitors induce quinate accumulation in plant leaves [7]. This physiological effect has been implicated in the toxicity of these herbicides [7], which raises the question of whether quinate mediates the toxic effects or mimics the action of the herbicides. In the latter case, the exogenous application of this compound could potentially be used as an herbicide. Natural product-based compounds, such as quinate, are considered safer than synthetic herbicides because of their relatively shorter persistence in the environment than that of synthetic herbicides [13,14], which is desirable from an environmental point of view. On the other hand, quinate is neither an EPSPS nor ALS-inhibitor, and its use would not increase the selection pressure on those action sites, which is important to avoid the development of resistance in weeds.

Foliar exogenous quinate application and quinate supply through the nutrient solution and root absorption have been tested on pea plants, and although both treatments have affected plant growth, only the residual application has been lethal [15]. More recently, the study of quinate foliar application has raised the possibility of the joint application of quinate and glyphosate to enhance glyphosate efficacy while lowering doses in the control of glyphosate-sensitive populations of *Amaranthus palmeri* [16]. Nevertheless, the potential herbicidal activity of quinate on different weed species has yet to be tested. Evaluating the applicability of quinate in weed control and understanding the physiology of treated plants might allow the adoption of quinate as an alternative herbicide for weed management practices.

The first objective of this work was to test the phytotoxicity of quinate on several common weeds. This was done by analyzing the effects of exogenous quinate application on the biomass of five representative weed species. Among the results obtained, a striking sensitivity of *Papaver rhoeas* to quinate was detected. This led the authors to the second objective of this study, which was to determine the suitability of quinate in the control of *P. rhoeas* populations that were sensitive and resistant to ALS-inhibitors, respectively, by evaluating the physiological effects of applying quinate or ALS-inhibiting herbicides.

## 2. Results and Discussion

### 2.1. Quinate Phytotoxicity Assay on Different Weed Species

In this study, the effect of quinate on six plant species of cereal agrosystems was studied to evaluate the phytotoxic and herbicidal activities of quinate. *Triticum aestivum* was used as a crop model to determine the phytotoxicity of quinate and was chosen because wheat is a widely-grown food crop [17]. Five target species were used as models of the most widespread weed families, including dicots and monocots, to obtain a full overview of the specific toxicity of quinate [18] with representatives of Poaceae and Crucifereae families, which includes the following problematic weeds [19]: *Cynodon dactylon, Bromus diandrus, Lolium rigidum, Sinapis alaba,* and *P. rhoeas.* This screening allowed us to achieve a wide response range and to not limit the assay to a particular weed species.

The parameter used in the biotest was the aboveground biomass 18 days after treatment, expressed as a percentage of the control treatment. The biotest showed that *L. rigidum, S. alba,* and *P. rhoeas* aboveground fresh biomass was 79%, 84%, and 12% of the control, respectively, and similar growth values were obtained in the dry weight per unit area (Table 1), which showed that their growth was affected by the presence of the quinate. The fresh weight of *T. aestivum* showed a nonsignificant decrease, while dry weight was not affected by quinate. There were no significant differences in the aboveground biomass of *T. aestivum, C. dactylon,* or *B. diandrus* (Table 1).

Although growth inhibition (measured as weight per area) due to quinate was similar in *L. rigidum* and in *S. alba* plants, the appearance of the dicot plants was more affected than the *L. rigidum* plants. Figure S1 shows the aspect of both species 7 days or 18 days after quinate application. One week after quinate treatment, the growth of treated *S. alba* plants was visually affected. The margins of the *S. alba* leaves got burned after quinate application, while no such symptoms were detected in the treated *L. rigidum* plants. So, the study showed that the two tested dicotyledonous weeds were more sensitive to the application of quinate than the examined monocotyledonous plants. Monocots are often less sensitive than dicots, similar to other organic compounds, such as the allelochemicals juglone [20] and benzoxazinoids [21].

*P. rhoeas* was, by far, the most sensitive weed to the quinate treatment. Indeed, other organic compounds, such as the allelochemical Dopa, have been reported to control field poppy growth as effectively as herbicides [22]. Corn poppy (*Papaver rhoeas* L.) is a problematic weed species, infesting extensive winter cereals that can decrease wheat yields by up to 32% [23]. The weediness of this competitive plant is due to an extended period of germination, high seed production, and a highly persistent seed-bank [24,25]. The high sensitivity of *P. rhoeas* to quinate allows for the option to use this organic compound in the control of this problematic weed.

### 2.2. Dose-Response Assay of Quinate on P. rhoeas

The application of quinate (400 mM) onto four true leaves containing *P. rhoeas* seedlings was lethal and decreased the aboveground biomass until 12% of the seedlings’ biomass remained. Nevertheless, to allow for the possibility of decreasing the dose of quinate applied for the control of corn poppy growth, a dose-response assay was performed. Further, the sensitivity of plants to organic compounds depends on plant age, so it remained to be determined if the sensitivity of *P. rhoeas* would be similar if quinate was applied to bigger plants.

**Figure 1:**
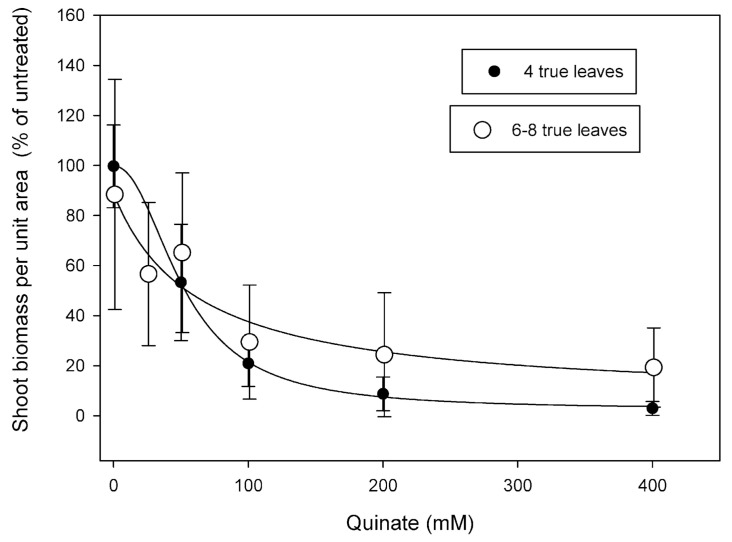
Dose-response of the shoot fresh biomass in *Papaver rhoeas* plants 18 days following quinate application, as a percentage of the control plants. Quinate was applied when plants showed four true leaves (black) or when plants had 6–8 true leaves (white). Log-logistic dose-response curves.

The dose-response experiments were performed with plants at two different phenological stages (4 and 6–8 true leaves), treated with five quinate concentrations (25, 50, 100, 200, and 400 mM) or treated only with the surfactant. The aspect of the plants 18 days after quinate treatment is shown in Figure S2. Figure 1 shows the effect on fresh weight per unit area, expressed as a percentage of the control plants. Although the fresh weight was highly variable within each dose, the effect on each phenological stage was described by a significant four-parameter log-logistic dose-response curve, and parameters of the two equations are shown in Table 2. The quinate concentration that reduced the shoot weight by 50% (ED_50_) was 52 mM for four true leaves and 62 mM for 6–8 true leaves, showing a decrease of sensitivity in bigger plants.

After the dose study, investigating the applicability and herbicidal activity of one dose lower than 400 mM but higher than the doses to be applied to inhibit corn poppy growth by 50% was required. As the ED_50_ was found to be 62 mM at the 6–8 true leaves stage, the phytotoxic effects of 100 mM quinate were evaluated.

### 2.3. Quinate or Sulfonylurea Herbicide Application on Sensitive and Resistant P. rhoeas Populations

*P. rhoeas* is a major weed affecting cereal crops in Europe and is a growing problem due to the appearance of herbicide-resistant biotypes to synthetic auxins and/or ALS-inhibitors, the latter due to the repeated use of sulfonylurea herbicides over time [26]. In most ALS-inhibitor-resistant cases studied, a target-site resistance mechanism has been identified [27], although several nontarget-site resistance mechanisms have been described over the last years [28].

Herbicides alone are not always enough to control herbicide-resistant corn poppy populations; therefore, the development of new management tools is required. Chemical control strategies should be combined with nonchemical ones. Different practical weed management strategies were tested to control sensitive and resistant populations of *P. rhoeas* to ALS inhibitors, such as tillage systems or integrated weed management [29]. In Spain, delayed sowing and other nonchemical practices have shown their effectiveness in reducing corn poppy densities, but only when combined with other control methods like chemical control or cultivation [30]. By the moment, corn poppy resistant populations can be controlled by the application of herbicides with alternative target sites [31]; however, in the context of the present scenario, with no new target sites discovered in recent decades [32] and considering that some of the herbicides that are currently successful in controlling corn poppy will not be available in the future due to legal limitations, the possible use of quinate as an alternative treatment to control resistant corn poppy was very interesting, and it was evaluated physiologically.

One hundred millimolar of either quinate or a sulfonylurea herbicide (a commercial mixture of amidosulfuron, iodosulfuron, and mesosulfuron) at the recommended dose was applied to sensitive and sulfonylurea-resistant populations of *P. rhoeas* at the phenological stage of 6–8 true leaves to evaluate quinate relevancy from two points of view. On the one hand, the toxicity of quinate was evaluated by growth parameters and the typical physiological effects of sulfonylurea herbicides. On the other hand, the possible mode of action of quinate was studied by focusing on the effects on the shikimate pathway, which is the pathway the quinate acts upon [33].

#### 2.3.1. Physiological Effects of Quinate or Sulfonylurea Herbicide

##### Shoot Biomass and Chlorophyll Content

**Figure 2:**
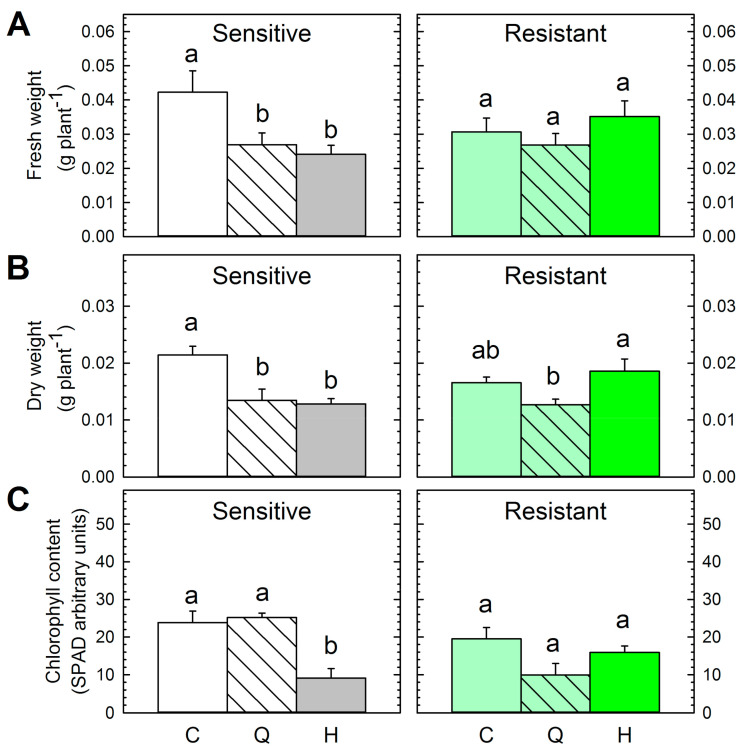
Fresh weight per plant (**A**), dry weight per plant (**B**), and chlorophyll content (**C**) in the sensitive (**left**) and resistant (**right**) *P. rhoeas* populations. Plants were untreated (Control, C) or treated with quinate (Q) or sulfonylurea herbicide (H) (Mean ± SE; *n* = 4–10). Different letters in each population indicate significant differences between treatments (*p*-value ≤ 0.05, Tukey).

The size of the untreated plants of both populations after 18 days was similar (Figure 2A,B). The effectiveness of herbicide treatment only on the sensitive population was confirmed by the repression of fresh and dry weight 18 days of herbicide application. As expected, the growth of resistant plants was not affected by the herbicide (Figure 2A,B).

Quinate treatment significantly decreased the fresh and dry biomass of the sensitive population compared with the untreated plants 18 days after treatment (Figure 2A,B). Although the decrease in the dry weight provoked by quinate in the resistant population was not statistically significant, a significant decrease was detected when comparing the effect of quinate with the effect of the herbicide on this population. These results suggest a potential role of quinate for the control of *P. rhoeas*-resistant populations, but further studies are needed to confirm the effectiveness of quinate in the control of resistant populations (Figure 2B). The drop in the total chlorophyll concentration was measured as a general stress marker three days after treatment application (Figure 2C). Only the sulfonylurea herbicide induced a decrease in the chlorophyll content on the sensitive population (Figure 2C). Amidosulfuron has been applied alone and shown to provoke a decrease in chlorophyll content in rapeseed in the same period of study (three days) [34]. ALS-inhibitors have been reported to induce growth arrest, photosynthesis inhibition, and to affect chlorophyll synthesis [35,36,37].

##### Physiological Parameters: Free Amino Acid Profile and Carbohydrate Content

**Figure 3:**
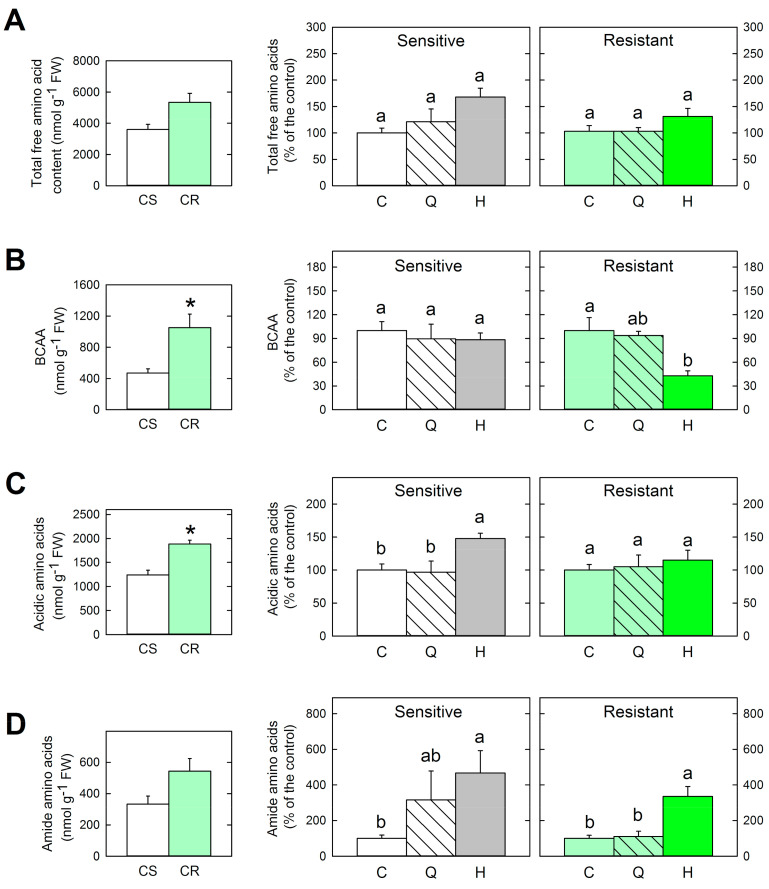
Amino acid profile. First column: Total free amino acids (**A**), branched-chain amino acid (BCAA) (**B**), acidic (Glu+Asp) (**C**), and amide (Gln+Asn) (**D**) contents in the untreated plants of the sensitive (CS) and resistant (CR) populations of *P. rhoeas*. The symbol (*) indicates significant differences between the control plants of each population (*p*-value ≤ 0.05, *t*-student). Second and third columns: amino acid profile of sensitive and resistant plants, respectively. Contents are expressed as % of the respective control. Plants were untreated (Control, C) or sampled three days after quinate (Q) or sulfonylurea herbicide (H) treatments (Mean ± SE; *n* = 4–5). Different letters in each population indicate significant differences between treatments (*p*-value ≤ 0.05, Tukey).

**Figure 4:**
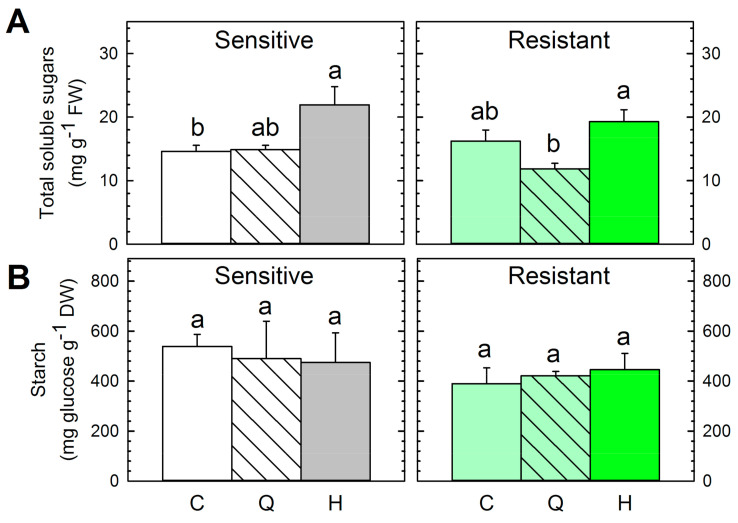
Total soluble sugars (**A**) and starch content (**B**) in the sensitive (**left**) and resistant (**right**) *P. rhoeas* populations. Plants were untreated (Control, C) or sampled three days after quinate (Q) or sulfonylurea herbicide (H) treatments (Mean ± SE; *n* = 4). Different letters in each population indicate significant differences between treatments (*p*-value ≤ 0.05, Tukey).

The effect of treatments on the most significant physiological parameters affected by sulfonylurea herbicides was evaluated: free amino acid profile (Figure 3) and carbohydrate content (Figure 4). The effects on the free amino acid profile were studied by evaluating four previously physiological parameters detected after ALS-inhibitors: total free amino acid content, branched-chain, and acidic and amide amino acid contents (Figure 3).

First, amino acid contents between control plants of both populations were compared (Figure 3). The total free amino acid pool and amide amino acid content were similar between untreated plants of both populations. Resistant plants showed a higher branched-chain amino acid (BCAA) and acidic amino acid contents. Previous works have described the accumulation of BCAA in the resistant populations of *Lactuca serriola* and *Solanum ptychanthum* [38,39]. Transgenic recombinant rice lines with mutated ALS genes have also shown BCAA accumulation [40]. It has been proposed that mutations at positions 197 and 574 do not drastically affect ALS functionality, but rather alter ALS sensitivity to BCAA feedback inhibition, resulting in the accumulation of these amino acids [41]. Indeed, mutations at positions 197 have been reported in resistant populations from Catalonia (Spain) [42], suggesting that this resistance mechanism would appear in the population used in this study.

As the differences between the basal levels of free amino acids of both populations would mask the specific changes in each population, the effects of the treatments on total amino acid content, BCAA, acidic, and amide amino acids were shown as their relative content to control values (Figure 3). After herbicide application, sensitive plants showed an increase in the total free amino acid content and the relative amide amino acid content (Figure 3A,D), which has been described in other species and after the application of ALS-inhibitors belonging to different chemical classes [4,7,43]; however, the previously described transitory decrease of BCAA and decrease in acidic amino acids provoked by ALS-inhibitors [7,43] were not detected (Figure 3B,C). As expected, the total free amino acid content was not affected by the herbicide in the resistant plants (Figure 3A). On the contrary, the amide amino acid content was increased in the resistant plants treated with the herbicide (Figure 3D). The BCAA content was more affected in the resistant population than in the sensitive population (Figure 3B), showing a decrease in the BCAA relative content, which can be related to a different BCAA feedback control in plants with a mutated ALS.

Quinate applied exogenously did not significantly affect the free amino acid profile. A similar trend was detected in the contents of amide and total amino acids of the sensitive population after quinate and herbicide treatments (Figure 3A,D). Previous studies have reported either no change or an increase in the total free amino acid content in different species sprayed with 400 mM of quinate [15,16].

Figure 4 shows the effect of the treatments on the carbohydrate content of both populations. Untreated plants showed similar content of the sum of total soluble sugars (glucose + fructose + sucrose) and starch (Figure 4). Accumulation of total soluble sugars in the sensitive plants treated with the ALS-inhibitor was detected (Figure 4A). This physiological effect can be considered as a typical marker of these herbicides as has been reported in other species, such as pea [5], *Arabidopsis thaliana* [44], and the weed *Amaranthus palmeri* [45]. This carbohydrate accumulation detected in the leaves after herbicide treatment has been previously attributed to growth arrest. The accumulation of carbohydrates in sinks abolishes the sugar gradient required for long-distance transport, and carbohydrates accumulate in the leaves of treated plants because of a decrease in sink strength [5]. Interestingly, the accumulation of total soluble sugars was not detected in the resistant population (Figure 4B). Carbohydrate accumulation was found in response to the application of the herbicide only in the sensitive population and not in the resistant one, which confirms that this effect can be considered as a physiological marker of herbicide toxicity.

Quinate treatment did not affect the carbohydrate content of sensitive plants, as has been reported before in pea [15]. The content of total soluble sugars in the resistant population decreased after the quinate supply, a decrease that was significant if compared with herbicide-treated plants (Figure 4A). This pattern suggests a secondary effect of quinate supply beyond the shikimate pathway (where the compound is incorporated) only in the resistant population, which would be related to the pleiotropic effects of the resistance mechanism.

#### 2.3.2. Approach to the Mode of Action of Quinate: Effects on the Shikimate Pathway

**Figure 5:**
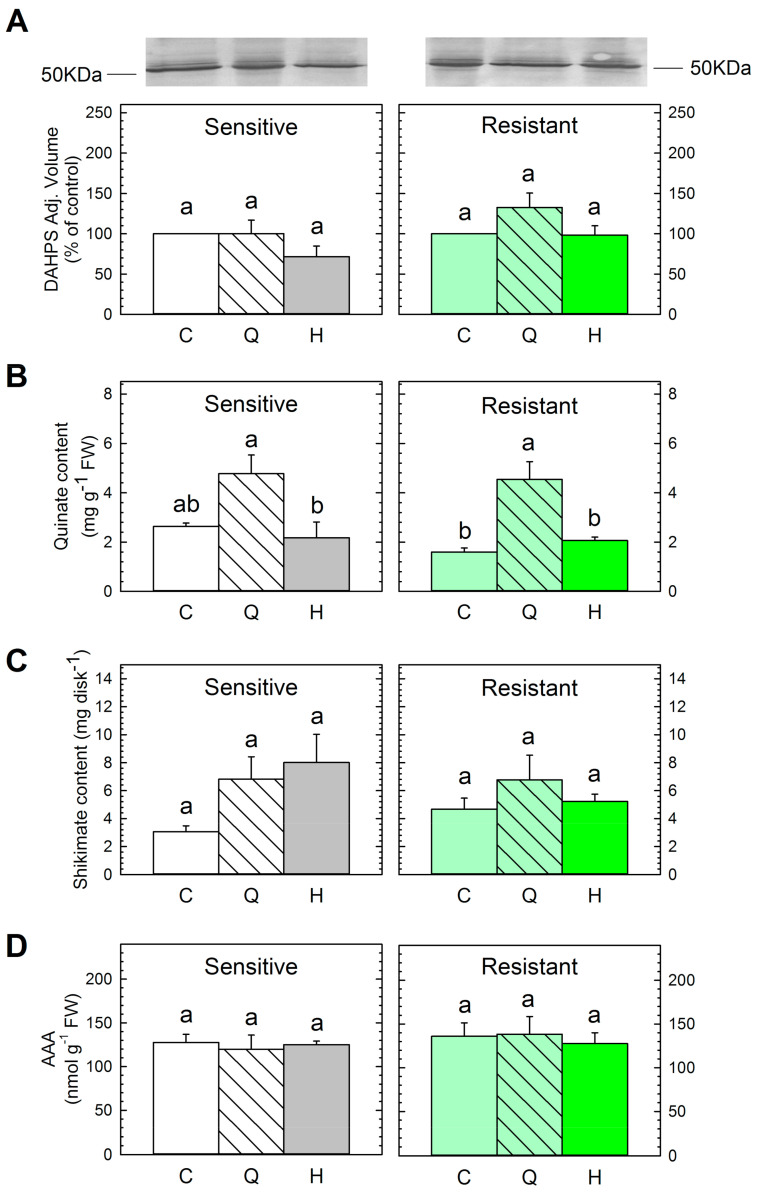
The pattern of the shikimate pathway. Plants were untreated (Control, C) or sampled three days after quinate (Q) or sulfonylurea herbicide (H) treatments. 3-deoxy-darabino-heptulosonate-7-phosphate-synthase (DAHPS) protein content (**A**). Band intensity on blots is presented as the relative ratio of the respective control (mean ± SE; *n* = 3). Control is arbitrarily presented as 100%. A representative blot is shown. Quinate (**B**), shikimate contents (**C**), and aromatic amino acid (AAA) content (**D**) are expressed as a percentage of the respective control (Mean ± SE; *n* = 4). Different letters in each population indicate significant differences between treatments (*p*-value ≤ 0.05, Tukey).

Table 1 and Figure 1 show the phytotoxic effect of quinate on several weed species, but the mode of action of the compound remains to be determined. We hypothesized that quinate toxicity would be mediated by the deregulation of the shikimate pathway—the pathway that quinate is incorporated into. The content of several metabolites and key enzymes of the shikimate pathway were determined in both populations (Figure 5). Shikimate was measured as an intermediate of the pathway, aromatic amino acid (AAA) content as final products, and 3-deoxy-darabino-heptulosonate-7-phosphate-synthase (DAHPS) as a key enzyme. The importance of the DAHPS protein is based on its control of the entrance of carbon flux to the AAA pathway [46,47].

The control plants of both populations showed similar DAHPS protein, quinate, shikimate, and AAA contents (Figure 5A–D). The AAA content of the untreated plants of both populations was similar (data not shown). These results suggest that the resistance mechanism has no pleiotropic effects on the shikimate pathway of the resistant population.

The DAHPS content was not affected by the herbicide in any of the populations (Figure 5A), which conforms to reports of the same effect in other ALS-inhibitors in *Amaranthus palmeri* leaves [45]. On the contrary, previous studies have shown induction of the secondary metabolism after treatment with ALS-inhibitors, which is related to an induction of the first enzyme of the shikimate pathway in pea roots [48]. Similarly, the sulfonylurea application did not induce quinate accumulation or AAA accumulation, as reported after other ALS-inhibitors [7,45]. The lack of detection of these typical physiological effects on the secondary metabolism after ALS-inhibition suggests that the study did not continue long enough to induce them.

The application of quinate to leaves increased the concentration of quinate in the leaves of both populations, although it was only significant in the resistant population. Quinate accumulation confirmed that the compound was absorbed (Figure 5B), as reported in other species [16,44]. No changes were detected in the DAHPS protein or shikimate content after quinate supply, as reported before [16]. Quinate has been proposed to serve as a carbon source for the biosynthesis of AAAs [33,49], and other studies have reported a general increase in the percentage of AAAs [15] or specific increases of specific AAAs after quinate supply [16,50]. Nevertheless, no specific changes in any particular AAA (data not shown) or in the general AAA content (Figure 5D) were detected in corn poppy plants after quinate supply. This lack of effect can be related to the low dose applied (the other studies have been performed with 400 mM, whereas in this study, 100 mM quinate was applied).

When quinate is supplied to the nutrient solution of pea roots, it has been shown to incorporate into the shikimate pathway and accumulated in the final products, such as lignin, concomitant with a decrease in the amount of DAHPS protein [50]. Quinate spray applied to the *A. palmeri* leaves has been incorporated and accumulated in tyrosine and phenylalanine [16]. Contrary to these previous studies, no effects on the shikimate pathway were detected in resistant or sensitive *P. rhoeas* populations after quinate treatment, suggesting that the phytotoxicity detected (Figure 1 and Figure 2) was not related to any specific change in the shikimate pathway in the short term.

The growth of the populations was affected by quinate concentrations from 100 mM (Figure 1 and Figure 2A,B), providing evidence of the phytotoxic effect of the quinate. Few changes were detected in the physiology of the populations three days after quinate treatment and were different between sensitive and resistant populations: a nonsignificant amino acid accumulation and a decrease in the carbohydrate content, respectively. It can be proposed that the mode of action of quinate is not related to changes in the shikimate pathway, and it is related to plant-specific perturbations in the carbon/nitrogen metabolism.

This study covers the gap between studies on the characterization of herbicide-resistant biotypes and those focused on the search for alternative control methods. On the one hand, the physiological response of herbicide-resistant biotypes to compounds of an organic nature, such as quinate, is approached. On the other hand, it opens a new scenario related to the phytotoxic effects of quinate and its use as a potential herbicide. Future research about optimizing the use of quinate as a potential herbicide will be necessary to unravel if more weeds apart from corn poppy are affected. The maximum concentration applied in this study—400 mM corresponds to a dose of 46 kg ha^−1^, and 100 mM corresponds to a dose of 11.5 kg ha^−1^. Both doses are too high and unrealistic, both from an economical and agronomic point of view. In this context, future experiments in order to improve quinate absorption by plant leaves would be necessary to decrease the dose per hectare. Additionally, from another point of view, high availability and low price of the synthesized compound would be necessary to achieve commercial development of quinate as an herbicide.

Recently it has been proposed the interesting possibility of the joint application of quinate and glyphosate to enhance glyphosate efficacy while lowering doses in the control of *Amaranthus palmeri* [16]. The combined application of quinate and a commercial herbicide can be proposed as an alternative use. In this context, the combined application of quinate and ALS-inhibitors would offer the possibility of controlling sensitive and resistant populations with the same application, although future experiments are needed in this direction.

## 3. Concluding Remarks

The phytotoxic effects of quinate reported in this study suggest that it may be a potential active compound for the development of alternative herbicides based on natural products. Among the weed species tested, the results show that quinate can be applied to control the growth of *P. rhoeas* onto 6–8 true leaves-seedlings and at concentrations above 100 mM.

The physiological characteristics of plants exposed to quinate have provided insights into its mode of action, which is more related to general perturbations in the carbon/nitrogen metabolism than to specific changes in the shikimate pathway.

This study lays the framework for the use of quinate in the control of sensitive and resistant populations of *P. rhoeas* to ALS-inhibitors, although future experiments are needed to optimize the quinate doses and to establish the mode of action of this potential natural herbicide.

## 4. Materials and Methods

### 4.1. Plant Material, Growing Conditions, and Treatments

The phytotoxicity of quinate was evaluated on five weed species. Plants of *Sinapis alba* L. and *Papaver rhoeas* L. were chosen as dicotyledon standard target species and *Bromus diandrus* Roth, *Cynodon dactylon* L., and *Lolium rigidum* Gaudin as monocotyledon standard target species. These species cover the most widespread weed families (Poaceae and Cruciferae) [19]. Wheat was used to checking the effects of the quinate, as it is a good representation of a widely-used standard crop.

Seeds of *S. alba, P. rhoeas, C. dactylon,* and *L. rigidum* were purchased from a seed commercializer (Semillas Silvestres S.L., Cordoba, Spain). Seeds of a *P. rhoeas* population, previously described as sulfonylurea-resistant, were kindly provided by J.M. Montull, University of Lleida, Lleida, Spain. Seeds had a planting density of 2640, 19,000, 6600, and 10,500 seeds m^−2^ of *S. alba, P. rhoeas, C. dactylon,* and *L. rigidum*, respectively. Seeds were scattered randomly using a plastic bottle with a holed cap. They were sown in trays (50 × 35 × 7 cm), filled with a mixture of potting soil:perlite (1:1). After sowing, trays were supplemented with 0.3% (p/v) gibberellin GA_3_.

Seeds of *B. diandrus* were kindly provided by J.M. Montull, University of Lleida, Lleida, Spain. *Bromus* seeds were sterilized in hypochlorite and HCl [51] and were sown in Petri dishes with 0.6% agar supplemented with 1 mM gibberellin GA_3_. Seeds were placed in a growth chamber at 22 °C/12 °C day/night and a 14 h photoperiod. After 4 days, seedlings were transplanted to the same trays as the other tested species to achieve a planting density of 400 plants m^−2^.

All trays were placed in a greenhouse in Pamplona (Spain) and watered daily to field capacity as needed using overhead irrigation.

Commercial quinate (analytical standard, 98% purity) was purchased from Sigma-Aldrich (Merck Life Science S.L.U., Madrid, Spain). The sulfonylurea herbicide applied was Pacifica Plus (5% amidosulfuron, 1% iodosulfuron-methyl-sodium, 3% mesosulfuron-methyl; Bayer CropScience, Valencia, Spain).

### 4.2. Quinate Phytotoxic Effect on Different Weeds

Quinate was applied to the leaves at a concentration of 400 mM in 5.4% of the adjuvant sodium lauryl sulfate (commercial formula Biopower 27.65% (p/v) (Bayer Crop Science, Madrid, Spain) [15,16]. This concentration was used as this was established as the maximum solubility of quinate in the spray mix (the adjuvant dissolved in water) without any subsequent precipitation on the leaf surface [15]. Control plants were sprayed with adjuvant only. Treatments were applied at water rate per area of 600 L ha ^−1^ using an aerograph (model Definik; Sagola) connected to a compressor (model Werther one, Breverrato, 60 W; 10 L m^−1^; 2.5 bar). Each tray represented one experimental unit. For each species, quinate was applied at the phenological stage or the height specified in the Results section (Table 1). Five quinate trays and five control trays were included for each species, and the experiment was repeated twice. Eighteen days after treatment, plants were harvested (above ground), and fresh weight was annotated. The dry weight (70 °C for 48 h) was measured.

### 4.3. Quinate Dose-Response Assay with P. rhoeas

A dose-response study was performed to verify the sensitivity of corn poppy plants. The dose-response relationship was established according to [52]. Trays with plants of uniform size and appearance were selected and were divided into two groups. Plants of each group were treated at a different phenological stage: when plants had 4 or 6–8 and true leaves. Within each group, the effect of increasing doses of quinate up to 400 mM was evaluated (25, 50, 100, 200, and 400 mM), using one half-tray as one experimental unit. The control plants were treated with the adjuvant. For biomass evaluation, the shoot fresh weights of each tray were determined 18 days after treatment. The experiment was repeated twice.

### 4.4. Physiological Study on Sensitive and Resistant P. rhoeas Populations

After the quinate dose-response study, a physiological study was performed by comparing the effect of the 100 mM dose and the sulfonylurea herbicide on each population of *P. rhoeas* (sensitive and resistant). The sulfonylurea herbicide was applied at the recommended field rate of the commercial product (400 g ha^−1^) with the same adjuvant as quinate. Control (untreated) plants were sprayed with the adjuvant alone. Treatments were performed using an aerograph (Junior Start model; Definik; Sagola, Vitoria-Gasteiz, Spain) and were applied when plants had 6–8 true leaves.

Three days after the onset of the treatment, the chlorophyll content was measured, and the leaves were harvested. At harvest, samples were obtained and immediately frozen in liquid nitrogen and stored at −80 °C for analytical determinations. Frozen samples were ground to a fine powder under liquid N_2_ using a Retsch mixer mill (MM200, Retsch GmbH, Haan, Germany), separately maintaining individual plants as biological repeats. The amount of tissue needed for each analysis was separated and stored at −80 °C. In each population, plants of each treatment were left for biomass determination after 18 days of treatment. The experiment was repeated twice.

#### 4.4.1. Biomass

The effect of 18 days of treatments on corn poppy biomass was determined by the evaluation of shoot fresh weight per plant. Samples were dried at 70 °C for 48 h, and the dry weights per plant were measured.

#### 4.4.2. Chlorophyll Content

Chlorophyll content in intact plants was measured in the youngest expanded leaf with Minolta SPAD-502 (Minolta Camera Co. Ltd., Osaka, Japan) chlorophyll meter.

#### 4.4.3. Analytical Determinations

1. Amino acid content: Amino acid content was determined in the same extracts used for quinate determination using capillary electrophoresis equipped with a laser-induced fluorescence detector, as previously described [7].
2. Carbohydrate content: The soluble carbohydrate (glucose, fructose, and sucrose) content was determined in ethanol-soluble extracts, and the ethanol-insoluble residue was extracted for starch analysis [5]. Carbohydrate levels were analyzed by ion chromatography in a 940 Professional IC Vario 2 instrument (Metrohm AG; Herisau: Switzerland) equipped with a Metrosep Carb2 guard 4.0 + Metrosep Carb2 150/4.0 column (Metrohm AG; Herisau: Suiza) at 30 °C. The eluent was 300 mM NaOH and 1 mM sodium acetate at a flow rate of 0.5 mL min^−1^. The detection was performed by amperometry.
3. DAHPS content: Proteins were separated by 12.5% SDS-PAGE, and the immunoblots were produced according to standard techniques. DAHPS immunoblotting was performed, as described previously [53].
4. Shikimate content: The determination of the content of shikimate in leaf disks of treated plants was determined spectrophotometrically, as described previously [43].
5. Quinate content: The 50 mg of tissue was extracted in a mixture of water:chloroform:methanol, as described before [54]. Quinate levels were analyzed by ion chromatography, as described before [16].

### 4.5. Statistical Analysis

For each plant species, a comparison between control and quinate treatments was assayed by the Student’s *t*-test, calculating mean values for shoot fresh and dry weight. For the evaluation of different quinate doses on corn poppy, dose-response curves based on fresh weight per unit area were constructed using the program Sigma Plot 14.0 (Systat Software, Inc., San Jose, CA, USA) to calculate the four-parameter sigmoidal log-logistic dose-response mode by the equation:

Y = C + ((D − C)/[1 + (x/ED_50_)^b^])

where y is the shoot biomass as % of the untreated, *x* is quinate concentration, C and D are the lower and upper limits of the curve, respectively, *b* is the slope, and ED_50_ is the herbicide dose or concentration resulting in 50% growth reduction.

In the physiological study, each mean value was calculated using samples from different individual plants from the two performed experiments as replicates. The difference between control plants of each population was evaluated using Student’s *t*-test and confirmed as significant when *p* < 0.05. The results of each population were subjected to separate one-way ANOVA analysis (SPSS 18.0), and the means were separated using the Tukey method (*p* < 0.05). For each population, significant differences are highlighted in the figures by different letters. Statistical analyses were performed using SPSS Statistics 25.0 (IBM, Corp., Armonk, NY, USA).

## Figures and Tables

**Table 1:** Effect of quinate (400 mM) applied to different plant species at the phenological stage or height indicated. Biomass parameters are expressed as a % of the biomass on control plants (only surfactant). Mean ± SE; *n* = 5.

Species	Phenological Stage at Quinate Application		Fresh Weight/m^2^ (% of Control)	Dry Weight/m^2^ (% of Control)
	Zadok Scale	Height		
*Triticum aestivum*	Z 12	8–10 cm	73.6 ± 8.6	92.8 ± 12.4
*Cynodon dactylon*	Z 12	5 cm	103.2 ± 24.1	115.9 ± 37.3
	Z 12	6–8 cm	82.1 ± 22.2	71.7 ± 16.4
*Bromus diandrus*	Z 13	10 cm	78.7 ± 6.5 ^1^	75.4 ± 4.7 ^1^
	2 true leaves		84.1 ± 3.7 ^1^	85.5 ± 4.9 ^1^
*Lolium rigidum*	4 true leaves		**12.5 ± 4.4 ^1^**	**12.3 ± 4.0 ^1^**
*Sinapis alba*				
*Papaver rhoeas*				

^1^ Statistically significant differences between quinate and control treatments (Student’s *t*-test, *p* < 0.05).

**Table 2:** Log-logistic parameters of the dose-response of the shoot fresh biomass in *Papaver rhoeas* plants 18 days following quinate application. Equation: Y = C + ((D − C)/[1 + (x/ED_50_)^b^]). C and D are the lower and upper limits of the curve, respectively, *b* is the slope, and ED_50_ is the herbicide dose or concentration resulting in a 50% growth reduction. *p*-values indicate the level of significance of curves fitting.

Phenological Stage at Quinate Application	Lower Limit	Upper Limit	Slope	ED_50_	ANOVA *p*-Value
4 true leaves	2.17	97.1	2.58	51.94	<0.0001
6–8 leaves	1.00	90.4	8.53	62.25	0.0077

## Supplementary Materials

The following are available online at https://www.mdpi.com/2223-7747/9/9/1215/s1, Figure S1: Aspect of the *Lolium rigidum* and *Sinapis alba* plants 7 days or 18 days after quinate application. Figure S2: Aspect of the *Papaver rhoeas* plants 18 days after treatment.
