# Supplementary material for: Physiological Approach to the Use of the Natural Compound Quinate in the Control of Sensitive and Resistant Papaver rhoeas
Source: Plants (Basel). 2020 Sep 16;9(9):1215. doi: 10.3390/plants9091215 (PMC7569983; doi:10.3390/plants9091215)
Supplement: Supplementary file 1 [file plants-09-01215-s001.pdf]

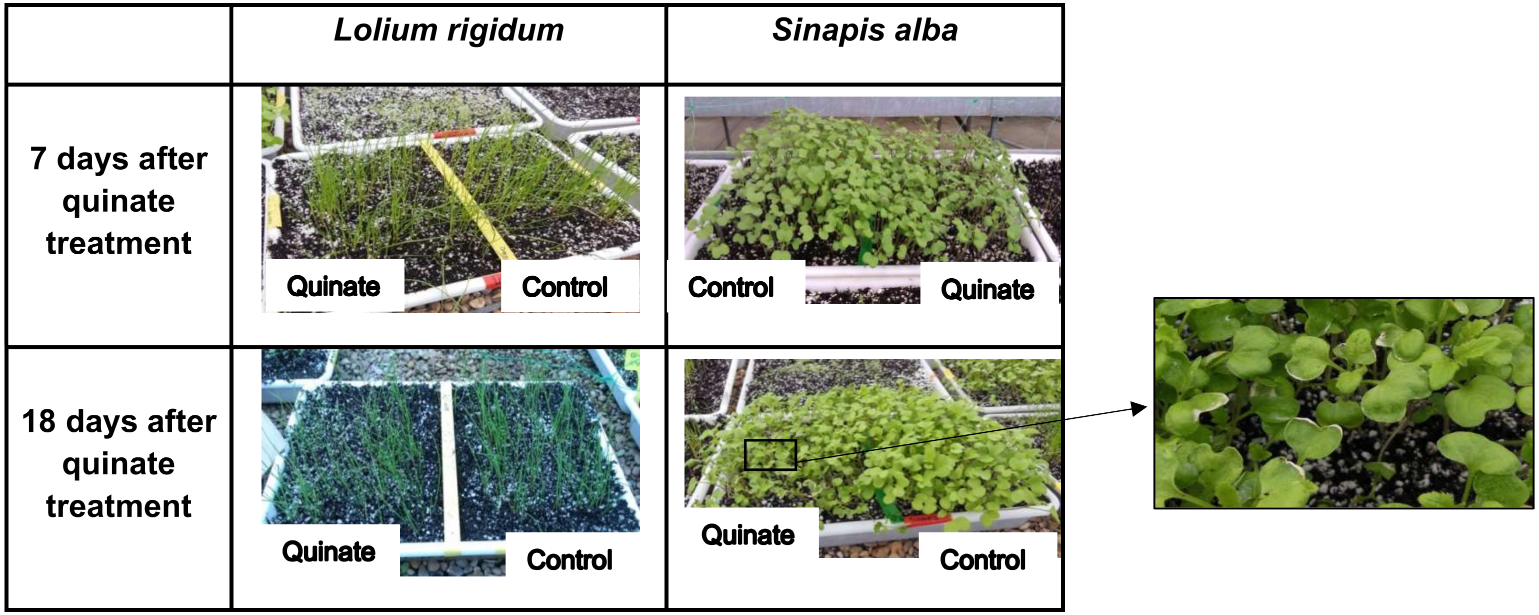

Figure S1: Aspect of the *Lolium rigidum* and *Sinapis alba* plants 7 and 18 days after treatment. 400 mM quinate was applied. Control plants were treated with only surfactant. The image in the box shows leaf border aspect of quinate-treated *S. alba* plants after 18 days.

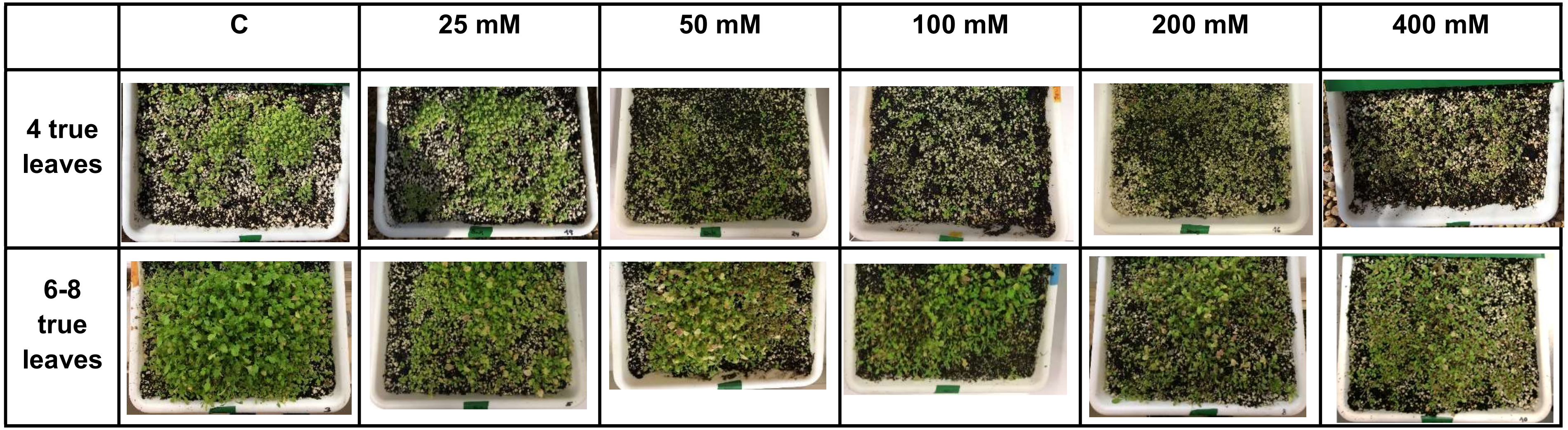

Figure S2: Aspect of the *Papaver rhoeas* plants 18 after treatment. Quinate (25-400 mM) was applied at two different phenological stages. Control plants were treated with only surfactant.
